# The Combination of Seven Preoperative Markers for Predicting Patients with Gastric Cancer to Be Either Stage IV or Non-Stage IV

**DOI:** 10.1155/2018/3450981

**Published:** 2018-06-03

**Authors:** Wei Ge, Li-ming Zheng, Gang Chen

**Affiliations:** Department of General Surgery, Nanjing Drum Tower Hospital, The Affiliated Hospital of Nanjing University Medical School, Nanjing, Jiangsu Province 210008, China

## Abstract

To assess whether preoperative markers could predict the stage of patients with gastric cancer. We analyzed retrospectively the preoperative indicators between stage IV and non-stage IV gastric cancer at the Gastrointestinal Surgery of Nanjing Drum Tower Hospital. A total of 500 patients with gastric cancer were screened. Of all the variables, alpha-fetoprotein (AFP), carcinoembryonic antigen (CEA), carbohydrate antigen (CA) 125, carbohydrate antigen (CA) 199, carbohydrate antigen (CA) 724, carbohydrate antigen (CA) 242, thrombin time (TT), prothrombin time (PT), activated partial thromboplastin time (APTT), blood platelet count (PLT), white blood cell (WBC) count, C-reactive protein (CRP), neutrophil count (NC), lymphocyte count (LC), neutrophil-lymphocyte ratio (NLR), hemoglobin (HB), aspartate aminotransferase (AST), and ascites were found to have statistical differences between the two groups. Then, Stepwise Discriminant Analysis was conducted to establish a prediction model including 7 indexes (CA724, CA242, TT, PLT, CRP, AST, and ascites). According to the model, 90.6% of original grouped cases were correctly classified and 90.6% of cross-validated grouped cases were correctly classified. We built a discriminant including CA724, CA242, TT, PLT, CRP, AST, and ascites for predicting patients with gastric cancer to be either stage IV or non-stage IV. According to this discriminant, 90.6% of patients could be correctly predicted.

## 1. Introduction

Gastric cancer is the second leading cause of cancer deaths worldwide despite a dramatic decline in its incidence over the past century [1]. Surgical resection of the primary tumor and regional lymph nodes is the only curative approach for gastric cancer. However, the overall prognosis remains poor and some cases are diagnosed as stage IV when seeing a doctor [2]. For patients with stage IV gastric cancer, surgery is not recommended except for patients with obstruction, perforation, or bleeding. So it is essential to assess the preoperative clinical stage to choose a proper treatment and avoid unnecessary surgery. At present, the assessment of the preoperative stage mainly relies on imaging examinations including computed tomography (CT), magnetic resonance imaging (MRI), and positron emission tomography-computed tomography (PET-CT). However, imaging examinations could not make an accurate diagnosis when the metastatic lesion is too small or too hidden. Thus, the patients may suffer unnecessary surgery when imaging cannot discover metastatic lesions. So we need to explore a new method to predict stage IV gastric cancer to make up for the inadequacy of imaging.

Stage IV gastric cancer affects other systems and leads to a change of many indexes such as coagulation, inflammation, and nutrition. These serum biomarkers have the potential to act as supplementary tools for predicting the preoperative stage of gastric cancer. In this study, we analyzed retrospectively the preoperative indicators between stage IV and non-stage IV gastric cancer to investigate whether these indicators could predict the stage preoperatively.

## 2. Materials and Methods

### 2.1. Patients

The patients who had an accurate stage for gastric cancer at the Department of General Surgery, the Affiliated Hospital of Nanjing University Medical School, between January 2014 and December 2016 were analyzed retrospectively. Inclusion criteria are as follows: (1) patients with complete medical records, (2) patients without operation or chemoradiotherapy previously, and (3) non-stage IV patients with pathological diagnosis and stage IV patients with pathological or imaging diagnosis. Reasons for exclusion include accompanying other tumors, thrombosis and hemorrhagic disease, viscera function disorder, diabetes mellitus, and acute inflammation. Patients with bone marrow involvement are also excluded. The patients were divided into stage IV and non-stage IV groups. The diagnosis of gastric cancer with stage IV is as follows: (1) patients with metastases of other organs such as the liver, lung, and pancreas; (2) patients with peritoneal spread; and (3) patients with metastases of distant lymph nodes such as the supraclavicular lymph nodes. This study was approved by the IRB of Nanjing Drum Tower Hospital, the Affiliated Hospital of Nanjing University Medical School. All methods were performed in accordance with the relevant guidelines and regulations. The written informed consents for participation in the study were obtained from all participants.

### 2.2. Analyzed Parameters

The parameters included in the analysis were categorized into clinical, imaging, and pathological data. The clinical indices included age, gender, tumor markers, coagulation function, inflammatory indices, nutritional status, and liver function. The imaging data was on whether the patient had ascites. The pathological data was mainly the TNM stage of gastric cancer. The pathological diagnosis was confirmed by at least two pathologists, and imaging diagnosis was also confirmed by at least two image doctors.

Tumor markers used in this analysis included alpha-fetoprotein (AFP), carcinoembryonic antigen (CEA), carbohydrate antigen (CA) 125, carbohydrate antigen (CA) 199, carbohydrate antigen (CA) 724, and carbohydrate antigen (CA) 242. These tumor markers were tested in the Nuclear Medicine Department through the tumor detection kit made by the Shanghai Biotechnology Co. Ltd. Coagulation function included activated partial thromboplastin time (APTT), prothrombin time (PT), thrombin time (TT), and blood platelet count (PLT). Inflammatory indices included white blood cell (WBC) count, neutrophil count (NC), lymphocyte count (LC), neutrophil-lymphocyte ratio (NLR), and C-reactive protein (CRP). Nutritional indices included hemoglobin (HB) and albumin (ALB). Liver enzyme parameters included alanine aminotransferase (ALT), aspartate aminotransferase (AST), total bilirubin (TBIL), and direct bilirubin (DBIL). TNM staging was performed according to the American Joint Committee on Cancer Staging Manual (7th edition).

### 2.3. Statistical Analysis

A large number of variables (22 variables) were investigated in this study. For comparisons, the chi-squared test and two-tailed Student *t*-test were performed to identify factors related to stage. Then we used the Stepwise Discriminant Analysis (SDA) to establish a simple and useful prediction model based on the significant predictors. At last, we got the accuracy through cross validation. This method does not need another validation cohort to validate the effect of the models. Results were expressed as a hazard ratio (HR) with 95% CI. A *p* value of less than 0.05 was considered to be statistically significant. All tests were two sided. Data analysis was done with the SPSS Version 19.0 software.

## 3. Results

### 3.1. Sociodemographic Characteristics of the Patients

500 patients were screened (354 men and 146 women, ages ranging from 21 to 92 years, with mean age 61.9 years). 470 patients were subjected to surgery. They were proved to be 128 cases of stage I, 101 cases of stage II, 172 cases of stage III, and 69 cases of stage IV gastric cancer. The remaining 30 cases were all diagnosed as stage IV by imaging examinations. The patients were divided into non-stage IV and stage IV groups. Stage I, stage II, and stage III were classified as the non-stage IV group. So there were 401 cases in the non-stage IV group and 99 cases in the stage IV group.  Table 1 showed the distributions of the sociodemographic characteristics of the two groups. No statistically significant differences were observed on gender and age. The cases in both groups were comparable, with good proportionality.

### 3.2. Screening for the Predictors Related to Stage

22 variables were analyzed in sequence, and the results are summarized in Table 1. We found that the following indexes had statistical differences between the two groups: AFP, CEA, CA125, CA199, CA724, CA242, TT, PT, APTT, PLT, WBC, CRP, NC, LC, NLR, HB, AST, and ascites.

### 3.3. Establishment of the Prediction Model

According to the screened 18 variables which were statistically significant, a prediction model of stage was conducted by a Stepwise Discriminant Analysis (*F*_Entry_ = 3.84, *F*_Removal_ = 2.71). The Stepwise Discriminant Analysis showed that Wilks lambda, as a test of the discriminant function, was significant (lambda = 0.506, chi-square = 318.809, df = 7, *p* ≤ 0.001). 7 variables were selected as follows: CA724, CA242, TT, PLT, CRP, AST, and ascites (Table 2). Then Classification Function Coefficients are shown in Table 3. According to Table 3, we got the Bayesian discriminants: *F*(non-stage IV) = 15.468 ∗ ascites − 0.001 ∗ CA724 + 0.001 ∗ CA242 + 9.366 ∗ TT + 0.044 ∗ PLT + 0.282 ∗ CRP − 0.035 ∗ AST − 97.274. *F*(stage IV) = 25.678 ∗ ascites − 0.001 ∗ CA724 + 0.002 ∗ CA242 + 9.091 ∗ TT + 0.048 ∗ PLT + 0.33 ∗ CRP + 0.002 ∗ AST − 108.563. For a new patient, we put the values of these 7 indexes into the two discriminants and calculated the discriminant function value. If the *F*(non-stage IV) > *F*(stage IV), the subject was considered to be non-stage IV, and if the *F*(non-stage IV) < *F*(stage IV), the subject was regarded as stage IV. Complementarily, for patients without ascites, the value of ascites was 1, while, for patients with ascites, the value of ascites was 2.

### 3.4. Predictive Effect Accuracy of the Prediction Model

The prediction of the accuracy of the prediction model was performed by cross validation (Table 4). The results showed that 90.6% of original grouped cases were correctly classified and 90.6% of cross-validated grouped cases were correctly classified.

## 4. Discussion

For patients with gastric cancer, preoperative tumor staging is useful to select the appropriate therapeutic strategy. Surgery is the main treatment measure for non-stage IV patients, while comprehensive therapy is proper for stage IV patients. So it is necessary to diagnose a patient as either stage IV or non-stage IV preoperatively. At present, the preoperative stage mainly relies on imaging examinations. However, imaging examinations easily lead to misdiagnosis or missed diagnosis when the metastatic lesion is too small or too hidden. Therefore, another method is needed to make up for the deficiency of imaging.

In the present study, we screened 7 indexes (CA724, CA242, TT, PLT, CRP, AST, and ascites) and got a discriminant for predicting stage. According to the cross validation, 90.6% of original grouped cases were correctly classified and 90.6% of cross-validated grouped cases were correctly classified. Specifically, 99.8% of the non-stage IV patients and 53.5% of the stage IV patients could be diagnosed by this discriminant. This discriminant combining with imaging could improve the accuracy of the preoperative stage of gastric cancer.

Tumor markers are often measured for early detection and preoperative staging of gastric cancer. The commonly used markers are AFP, CEA, CA125, CA199, CA724, and CA242. Some studies have suggested their relevance to predict the preoperative stage. Cidon and Bustamante showed that the preoperative serum level of CA724 has the best predictive value in indicating advanced disease in patients diagnosed with gastric cancer [3]. Chen et al. found that CA724 was the most correlative serum tumor marker for gastric cancer [4]. Li et al. evaluated the correlation between tumor markers and the lymph node metastasis of gastric cancer and found that CA724 and CA242 were evaluated significantly in the gastric patients with later N stage [5]. Jing et al. found that CA724 and CA242 combined with other markers were a good evaluation indictor for gastric cancer [6]. Thus, CA724 and CA242 play an important role in predicting the preoperative stage.

Studies showed that the host inflammatory response to cancer cells is associated with tumor progression [7, 8]. Inflammation-associated proteins have been found to increase in malignancies. CRP is one of the inflammatory markers, which was found to be associated with poor prognosis in kinds of solid tumors [9–11]. Kong et al. found that high serum CRP level was associated with aggressive pathological features and was an independent poor prognostic factor for recurrent gastric cancer, which might be a potential prognostic marker for recurrent gastric cancer patients [12]. A study by Wang et al. suggests that CRP was directly related to the severity of the gastric cancer and a combination of some inflammatory serum proteins including CRP may serve as noninvasive markers to assess the severity status and stage of gastric cancer [13]. In this study, we found that CRP could contribute to predict the stage of gastric cancer and built an exact discriminant formula including CRP to predict the stage.

AST is a critical enzyme during the biological process. AST may increase in different hepatic injures, such as hepatitis and cirrhosis induced by alcohol, drugs, viruses, and being under oxidative stress [14]. Oxidative stress and inflammation are associated with gastric cancer development, while oxidative stress and inflammation could also lead to the damage of liver cells. So AST may increase in gastric cancer. Reports have suggested that the presence of a systemic inflammatory response is linked to poor survival in patients with gastric cancer [15]. Therefore, AST could predict prognosis to some extent. Our study showed that the level of AST has significant difference between stage IV gastric cancer and non-stage IV and AST could help to predict the stage preoperatively.

Systemic hemostasis and thrombosis activation has been implicated in tumor progression and metastasis. The famous HYPERCAN study showed that hypercoagulation screening was an innovative tool for risk assessment, early diagnosis, and prognosis in cancer [16]. PLT and blood coagulation abnormalities occur frequently in gastric cancer. Some hematological parameters such as platelet have diagnostic power and can discriminate patients with gastric cancer from patients without cancer [17]. Thrombocytosis is considered an important risk factor, and it is associated with poor gastric cancer prognosis [18]. Chen et al. built the model “HALP” including PLT, which was closely associated with clinicopathological features and was an independent prognostic factor in gastric patients [19]. A series of studies showed that PLT could predict poor survival in patients with gastric cancer [20, 21]. Few researches showed that PLT and TT could take part in predicting the preoperative stage of gastric cancer. In our study, we found that PLT and TT could contribute to predicting stage.

For patients with advanced gastric cancer, preoperative examinations often revealed abnormal ascites, which could indicate the possibility of peritoneal metastasis. A Japanese research indicated that the presence of ascites was significantly correlated with peritoneal metastasis (*p* < 0.005), which was an independent prognostic factor. They concluded that the presence of ascites was strongly associated with peritoneal metastasis and might indicate the need for diagnostic laparoscopy to evaluate stage IV factors and select the best treatment strategy [22]. Pongpornsup et al. studied that peritoneal nodules, increased peritoneal fat density, ascites, and peritoneal thickening are ancillary signs suggestive of peritoneal carcinomatosis [23]. Repiso et al. also found that ascites are an important predictive factor of peritoneal carcinomatosis and may have significant implications in the management of patients with gastric cancer [24]. Similarly, there was a report that rapidly progressing ascites may be the sole presenting symptom of metastatic gastrointestinal carcinoma [25]. Besides, the survival of gastric cancer patients with ascites is relatively short, and the presence of ascites was an important prognostic factor [26]. Cheong et al. got a similar conclusion that the presence of ascites was closely associated with peritoneal metastasis and was the most significant independent prognostic factor in advanced gastric cancer [27]. Thus, ascites play an important role in predicting the stage and prognosis in gastric cancer. Our study showed that all patients with ascites were proved to be stage IV and ascites have a great effect in predicting the preoperative stage.

It is important to determine the stage preoperatively in order to choose the appropriate management of gastric cancer. The preoperative stage mainly relies on imaging examinations. Imaging examinations could not make a definite diagnosis when the metastatic lesion is too small or too hidden. So we need to explore new methods to make up for the inadequacy of imaging. However, there were few reports on this topic. Ohi et al. identified that a combination of specific factors is an alternative method to preoperatively discriminate patients with gastric cancer with peritoneal metastasis from those without [28]. This study focused on predicting peritoneal metastasis and on predicting patients with gastric cancer to be either stage IV or non-stage IV, which has more valuable clinical significance.

## 5. Conclusion

We built a discriminant including CA724, CA242, TT, PLT, CRP, AST, and ascites for predicting patients with gastric cancer to be either stage IV or non-stage IV. According to this discriminant, 90.6% of patients could be correctly predicted. We will also carry out a prospective study to verify whether this discriminant could be used clinically.

## Figures and Tables

**Table 1:** Comparison of the clinical indexes between stage IV and non-stage IV groups.

Indexes	Stage IV group	Non-stage IV group	*p* value
Gender			0.085
Male	63	291	
Female	36	110	
Age (yr)	61.29 ± 12.92	62.01 ± 11.24	0.802
AFP (ng/mL)	8.06 ± 29.37	3.5 ± 6.95	0.006
CEA (ng/mL)	130.50 ± 784.63	6.66 ± 36.10	0.002
CA125 (U/mL)	42.45 ± 55.44	10.36 ± 8.33	≤0.001
CA199 (U/mL)	522.09 ± 2187.79	43.35 ± 190.26	≤0.001
CA724 (U/mL)	372.37 ± 3129.16	7.21 ± 42.39	0.022
CA242 (U/mL)	473.32 ± 1925	31.30 ± 208.94	≤0.001
TT (s)	17.83 ± 1.57	18.38 ± 1.45	0.001
PT (s)	12.23 ± 0.88	11.91 ± 1.21	0.015
APTT (s)	29.77 ± 4.20	28.37 ± 4.00	0.002
PLT (∗10^9^/L)	233.19 ± 99.08	208.22 ± 69.68	0.004
WBC (∗10^9^/L)	6.02 ± 1.83	5.5 ± 1.52	0.005
CRP (mg/L)	12.41 ± 18.75	4.38 ± 4.78	≤0.001
NC (∗10^9^/L)	3.9 ± 1.58	3.24 ± 1.15	≤0.001
LC (∗10^9^/L)	1.49 ± 0.56	1.73 ± 0.62	0.001
NLR	3.10 ± 1.99	2.07 ± 1.05	≤0.001
HB (g/L)	113.45 ± 25.53	124.48 ± 25.53	≤0.001
ALB (g/L)	36.80 ± 4.05	39.18 ± 15.33	0.129
ALT (U/L)	21.30 ± 28.90	18.19 ± 10.31	0.08
AST (U/L)	24.89 ± 27.71	19.28 ± 6.98	≤0.001
TB (*μ*mol/L)	10.63 ± 4.94	11.27 ± 5.45	0.289
DB (*μ*mol/L)	3.67 ± 1.97	3.55 ± 1.86	0.586
Ascites			≤0.001
Yes	47	0	
No	52	401	

**Table 2:** The most significant variables discriminating between the groups by Stepwise Discriminant Analysis.

Indexes	*λ*	*F*	df1	df2	Sig.
CA724 (U/mL)	0.525	105.874	4	469	≤0.001
CA242 (U/mL)	0.551	192.145	2	471	≤0.001
TT (s)	0.511	74.545	6	467	≤0.001
PLT (∗10^9^/L)	0.506	64.897	7	466	≤0.001
CRP (mg/L)	0.537	134.919	3	470	≤0.001
AST (U/L)	0.519	86.86	5	468	≤0.001
Ascites	0.587	331.467	1	472	≤0.001

**Table 3:** The classification function coefficients.

	Stage	
	Non-stage IV group	Stage IV group
Ascites	15.468	25.678
CA724 (U/mL)	-0.001	−0.001
CA242 (U/mL)	0.001	0.002
TT (s)	9.366	9.091
PLT (∗10^9^/L)	0.044	0.048
CRP (mg/L)	0.282	0.33
AST (U/L)	−0.035	0.002
Constant	−97.274	−108.563

**Table 4:** The result of cross validation by using 7 variables.

Category			Groups prediction		Total
			Stage IV	Non-stage IV	
Original	Counting	Stage IV	53	46	99
		Non-stage IV	1	400	401
	%	Stage IV	53.5	46.5	100
		Non-stage IV	0.2	99.8	100

Cross validation	Counting	Stage IV	53	46	99
		Non-stage IV	1	400	401
	%	Stage IV	53.5	46.5	100
		Non-stage IV	0.2	99.8	100

Cross validation is done only for those cases in the analysis. In cross validation, each case is classified by the functions derived from all cases other than that case. 90.6% of the original grouped cases were correctly classified. 90.6% of the cross-validated grouped cases were correctly classified.
